# Occupancy modeling reveals territory-level effects of nest boxes on the presence, colonization, and persistence of a declining raptor in a fruit-growing region

**DOI:** 10.1371/journal.pone.0185701

**Published:** 2017-10-18

**Authors:** Megan E. Shave, Catherine A. Lindell

**Affiliations:** 1 Department of Integrative Biology, Michigan State University, East Lansing, Michigan, United States of America; 2 Program in Ecology, Evolutionary Biology and Behavior, Michigan State University, East Lansing, Michigan, United States of America; 3 Center for Global Change and Earth Observations, Michigan State University, East Lansing, Michigan, United States of America; Charles University, CZECH REPUBLIC

## Abstract

Nest boxes for predators in agricultural regions are an easily implemented tool to improve local habitat quality with potential benefits for both conservation and agriculture. The potential for nest boxes to increase raptor populations in agricultural regions is of particular interest given their positions as top predators. This study examined the effects of cherry orchard nest boxes on the local breeding population of a declining species, the American Kestrel (*Falco sparverius*), in a fruit-growing region of Michigan. During the 2013–2016 study, we added a total of 23 new nest boxes in addition to 24 intact boxes installed previously; kestrels used up to 100% of our new boxes each season. We conducted temporally-replicated surveys along four roadside transects divided into 1.6 km × 500 m sites. We developed a multi-season occupancy model under a Bayesian framework and found that nest boxes had strong positive effects on first-year site occupancy, site colonization, and site persistence probabilities. The estimated number of occupied sites increased between 2013 and 2016, which correlated with the increase in number of sites with boxes. Kestrel detections decreased with survey date but were not affected by time of day or activity at the boxes themselves. These results indicate that nest boxes determined the presence of kestrels at our study sites and support the conclusion that the local kestrel population is likely limited by nest site availability. Furthermore, our results are highly relevant to the farmers on whose properties the boxes were installed, for we can conclude that installing a nest box in an orchard resulted in a high probability of kestrels occupying that orchard or the areas adjacent to it.

## Introduction

Increases in anthropogenic land use, particularly agricultural expansion and intensification, pose a significant threat to many species and the functional diversity of species assemblages [1,2]. One form of habitat degradation imposed by human development is the loss of structural resources for nesting, such as cavities or platforms, which are essential for breeding and therefore important for the population dynamics of bird species [3]. Thus, loss of nest sites in human-modified habitats can negatively affect abundance and diversity of birds [4,5]. Agricultural intensification in particular removes mature trees that are important sources of nesting cavities [6], and reductions in cavity-bearing trees can limit cavity-nesting bird populations and assemblages [7,8]. However, many cavity-dependent birds species will use artificial cavities, such as nest boxes, in areas where natural cavities are scarce. Installing nest boxes in agroecosystems has increased populations of some species, such as the Eurasian Hoopoe (*Upupa epops*)[9], European Roller (*Coracias garrulus*)[10], and Eurasian Kestrel (*Falco tinnunculus*)[11]. Nest boxes could therefore play an important role in maintaining biodiversity in agroecosystems. Nest boxes may also benefit agriculture and agroforestry by increasing the strength of ecosystem services provided by predators, particularly increased predation on pest species [11–17].

Raptors are potentially important predators in agroecosystems based on their positions as top predators [12]. For example, American Kestrels (*Falco sparverius*; hereafter “kestrels”) are of interest as predators in fruit-growing regions because their diet typically consists of insects, small mammals, and birds [18] and can include a variety of orchard pests [19]. Furthermore, the American Kestrel is a species of conservation concern [20–22] that will readily use nest boxes in open areas with appropriate hunting habitat [18,23]. While previous research has demonstrated nest site limitation in kestrels, installing nest boxes will not always benefit populations, nor will monitoring nest box use alone reveal the effects of nest box installation on a local kestrel population [24]. Thus, the effectiveness of any nest box program can only be determined by considering other demographic parameters of the population. In addition, while previous studies have demonstrated that installing 100 or more nest boxes can increase the densities of American and Eurasian kestrels at a regional level of multiple km^2^ [8, 25], no previous studies have investigated the effect of nest boxes on kestrel presence at the level of potential kestrel territories. The effect of nest boxes on kestrel presence at this finer level in an agricultural region would provide useful information for farmers and other landowners on whose properties nest boxes are or will be installed. Measuring change in kestrel presence at the level of kestrel territories can capture the potential effect of individual landowners installing even one box each, thus informing landowners of whether their individual efforts will lead to increased kestrel presence on their properties. Finally, few studies have focused on kestrel populations in agroecosystems, and previous research has found inconclusive evidence of the effects of nest boxes on kestrel presence in fruit orchards [26].

**Fig 1:**
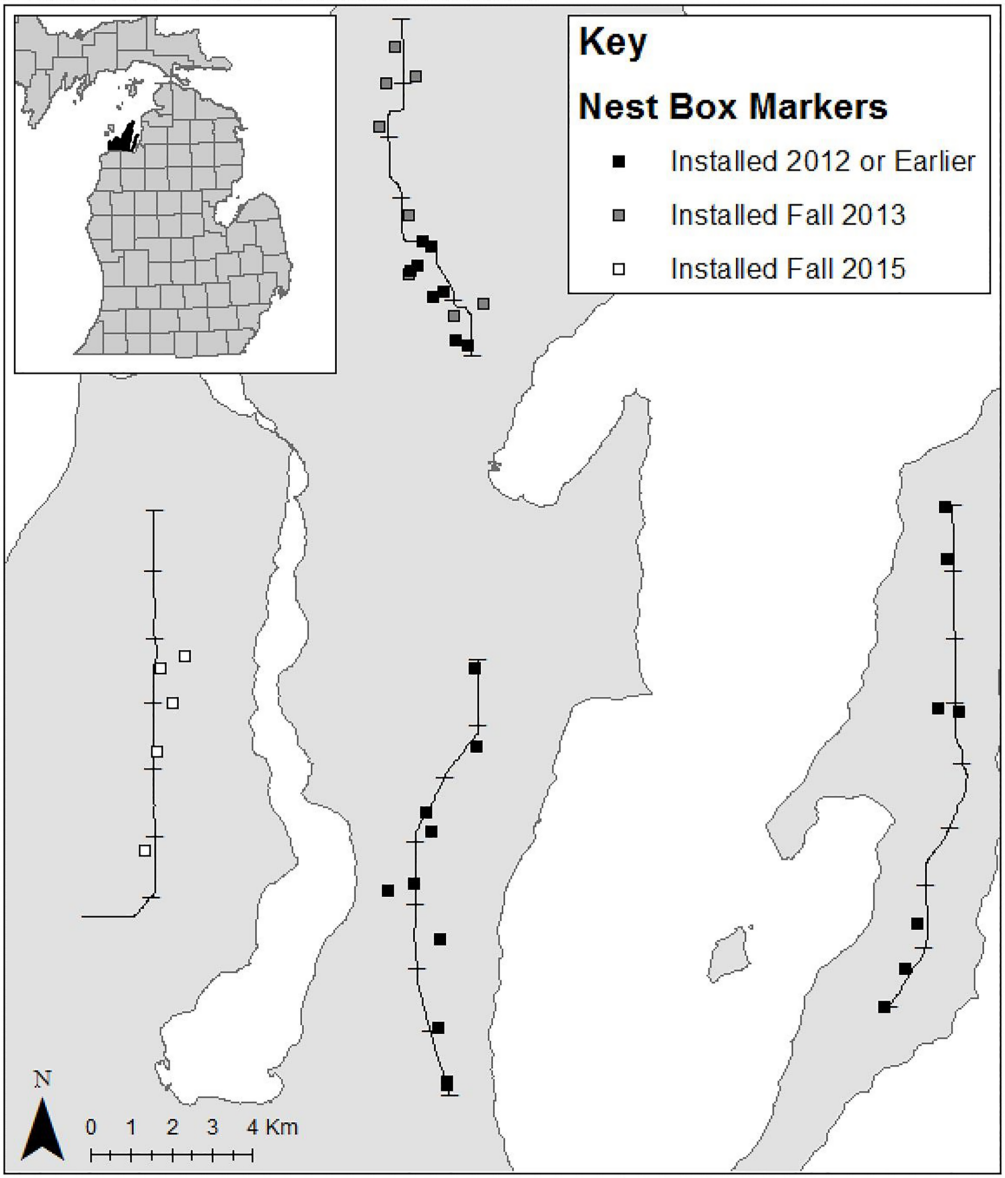
Map of survey sites in the northwestern Michigan study region (Leelanau and Old Mission Peninsulas). Lines indicate the four kestrel survey transects (divided into 28 1.6 km-long survey sites); markers indicate nest boxes located within 0.8 km of either side of transects. Inset: Map of Michigan with Leelanau and Old Mission Peninsulas shown in black.

This study used surveys of kestrel presence/absence and multi-season occupancy modeling to determine whether nest boxes in a fruit-growing region of northwestern Michigan (Fig 1) increased the presence of breeding kestrels, as defined by kestrel occupancy of potential territories (sites). While previous work has used occupancy modeling to investigate kestrel nest box use [27], this is the first study to use occupancy modeling to test the relative influence of nest boxes on kestrel site occupancy throughout a landscape. Our hypothesis was that this region lacks natural nesting cavities, thus the kestrel population is limited by nest cavity availability. We therefore predicted that sites (1.6 km × 500 m areas; 250 m on either side of a transect) with nest boxes would be more likely to be occupied by kestrels than sites without, and that increasing the number of sites with nest boxes would in turn increase the number of sites occupied by kestrels.

## Materials and methods

This research was approved by the Institutional Animal Care and Use Committee at Michigan State University (AUF #01/13-014-00). Private landowners granted us permission to conduct this study on their properties.

### Kestrel nest boxes in a fruit-growing region of Michigan

We conducted this study on the Leelanau and Old Mission Peninsulas of northwestern Michigan (45.0751°N–44.8365°N, 85.5032°W–85.7758°W), a major cherry-growing region that also produces apples and wine grapes, among other crops [28]. The region is largely agricultural with some residential and forested areas [28].

Kestrels are present in the study area during the summer breeding season; the kestrel population in this region is considered entirely migratory [18,29]. Populations in northern Michigan have declined since the 1960s [21,22]. Kestrel nest boxes have been present on utility poles in this region since the late 1980s (F. Otto, personal communication); 24 of these older boxes remained intact during this study. We installed a total of 23 new boxes (described in [30]) in cherry orchards during this study: 8 prior to the 2013 season, 10 prior to the 2014 season, and 5 prior to the 2016 season (Fig 1). During the 2013–2015 breeding seasons, kestrels used 93% of the available new boxes and showed 91% nesting success, with a mean of 4.0 ± 0.2 fledglings produced per nest [30]. While we did not regularly monitor the older nest boxes installed on utility poles, our opportunistic observations indicated that at least 41% were used by kestrels, and those that may have been unused were likely placed too close to another nest box for both to be occupied [30]. Kestrels using these boxes provisioned their young with prey types in the following proportions: 81.3% arthropods, 13.5% mammals, and 2.3% birds [19].

### Kestrel surveys

Our survey design consisted of 4 roadside transects (9.6 km– 12.8 km long) divided into 1.6 km survey sites (Fig 1). We placed the starting point of each transect randomly, but we purposefully placed transects along roads in predominately non-forested areas with high densities of cherry orchards, so that each survey site consisted of mostly open habitat with orchards, matching kestrel habitat preferences [31].

We conducted temporally-replicated surveys in 2013–2016 during the kestrel breeding season. The surveys began each year between 9–21 June, after most of the known kestrel nests had hatched [30], so that kestrels would no longer be incubating eggs and would therefore be available for detection. The surveys ended each year by 12 August, prior to fall migration; the kestrel population in this region is considered entirely migratory [18,29]. We attempted to conduct as many surveys as possible during the brood rearing period of the breeding season, with 96% of surveys conducted in June and July. We assumed temporal and geographical population closure for each year because kestrel pairs hold territories for the entire breeding season [18]; however, it is possible this assumption was violated by adults dispersing along with fledglings during the postfledging period prior to migration [32].

During a survey round, we surveyed a site on foot from south to north (initial survey), waited for 5 min, and then surveyed the site again from north to south (return survey). Each survey round therefore yielded 2 surveys. These sequential time intervals may violate the assumption of independent and equal detection probabilities among surveys within the survey season [33], but because the same observer conducted all surveys on foot, this practice was logistically cost-effective for obtaining more temporal replicates. We surveyed the same 28 sites within the same 4 transects in 2013–2016. We surveyed each site 6 times in 2013 and 12 times in 2014–2016. We conducted all surveys between 0830 and 1230 EST (AM period) or 1600 and 2000 EST (PM period) on days without precipitation or fog. During each survey we recorded whether we detected an adult kestrel within 250 m of either side of the transect.

### Occupancy modeling

We investigated the effects of nest boxes and other factors (detailed below) on kestrel presence and detectability using multi-season occupancy modeling under a Bayesian framework [34]. This modeling approach included the following ecological processes:

$$z_{i,1}∼Bernoulli\;(\Psi_{i,1})\;at\;initial\;state\;(t=1)$$

$$z_{i,t}∼Bernoulli\;(z_{i,(t\;-1)}\;\times \;\varphi_{i,t}+(1-\;z_{i,(t-1)})\;\times \;\gamma_{i,t})\;for\;years\;t>1$$

where the occurrence state *z*_*i*,*t*_ = 1 if at least one kestrel was present at site *i* in year *t*. The first year site occupancy probability *ψ*_*i*,*1*_ determined the initial occurrence state at site *i*; the site colonization probability γ_*i*,*t*_ and site persistence probability *φ*_*i*,*t*_ determined the occurrence state in subsequent years.

The multi-season occupancy model also included the following observation process:

$$y_{i,k,t}|z_{i}∼Bernoulli\;(z_{i,t\;}\times \;p_{i,k,t})$$

where detection state *y*_*i*,*k*,*t*_ = 1 if a kestrel was detected at site *i* during survey *k* in year *t*. The occurrence state *z*_*i*,*t*_ and the detection probability *p*_*i*,*k*,*t*_. determined whether a kestrel was detected at site *i* during survey *k* in year *t*.

We included the 28 segment sites as spatial replicates within the 4 transects. We included the transect level to account for the potential non-independence associated with using groups of sites along 4 roads (Fig 1; [33]).

#### Potential factors affecting kestrel presence, colonization, and persistence

We predicted that kestrel presence would be more likely at sites with nest boxes. We therefore characterized each survey site based on whether it had nest boxes within 0.8 km of the transect (*site boxes*). We chose this distance given that a typical kestrel home range is 0.5 km– 2.4 km in diameter [18,35] and that the typical kestrel nest box spacing recommendation is 0.8 km apart [23]; we therefore assumed that a kestrel pair using a nest box would have a home range that included a large proportion of the survey site. We searched the landscape within 0.8 km of either side of the transects each year in order to locate any nest boxes installed by other groups or landowners.

We also considered the potential spatial dependence of sites along a transect. We included transect ID to account for the possibility that kestrel presence was more similar at sites within the same transect compared to sites from other transects (*transect*) [33]. In addition, given the variation in placement of nest boxes along a transect (Fig 1), we predicted that some kestrel home ranges could overlap two neighboring sites. We therefore determined whether kestrel presence was more likely at a site adjacent to a site with a box compared to a site without boxes in adjacent sites (*neighbor box*).

We modeled the site occupancy, colonization, and persistence probabilities as follows:

$$logit\;(\Psi_{i,j,1})=\alpha_{0}+\;{transect}_{j}+\;\alpha_{1}({site\;box}_{i,j,1})+\;\alpha_{2}({neighbor\;box}_{i,j,1})$$

$$logit\;(\gamma_{i,j,t})=\alpha_{3}\;+{transect}_{j}+\alpha_{4}({site\;box}_{i,j,t})+\alpha_{5}({neighbor\;box}_{i,j,t})$$

$$logit\;(\varphi_{i,j,t})=\alpha_{6}\;+{transect}_{j}+\alpha_{7}({site\;box}_{i,j,t})+\alpha_{8}({neighbor\;box}_{i,j,t})$$

where *transect*_*j*_ represented the random effect of transect *j*, and α_*1*,*2*,*4*,*5*,*7*,*8*_ represented the logit-linear coefficients for model covariates [33].

#### Potential factors affecting kestrel detectability

We first considered the potential effects of survey timing on kestrel detections. Kestrels exhibit conspicuous hunting behaviors, such as hover-hunting and use of elevated, exposed perches [18]. We predicted that kestrel detections would be higher during the brood rearing period of the breeding season (June–July for kestrels using the nest boxes [30]) because this period should correspond to higher hunting activity due to nestling provisioning. We used Julian date (*date*) to determine whether kestrels became more or less conspicuous as the survey season progressed [36]. We also considered whether detection rates differed between AM and PM survey periods (*time*).

We also addressed a biological explanation underlying the potential temporal dependence between initial and return surveys of a site during a survey round. We investigated whether observer presence during an initial survey of a site influenced kestrel behavior and affected detection during the return survey (*survey*). We predicted that kestrels might avoid the survey area after the initial survey, thus decreasing the detection probability during return surveys.

Finally, kestrel activity at known nest sites can bias survey detections [25]. We attempted to install nest boxes more than 250 m from the transect in order to avoid this potential bias during our surveys of kestrel presence within 250 m of either side of a transect; however, orchard availability required us to install some boxes within 250 m of a transect, and some of the older boxes had been installed within 250 m. We instead addressed this potential source of bias in detectability by determining whether detection rates were higher when nest boxes with active nesting attempts were within the survey area. We therefore characterized each site based on whether it had an active nest box within 250 m of the transect (*nest distance*).

We modeled the detection probability as follows:

$$logit\;(p_{i,k,t})=\beta_{0}+\beta_{1}({date}_{i,k,t})+\beta_{2}({time}_{i,k,t})+\beta_{3}({survey}_{i,k,t})+\beta_{4}({nest\;distance}_{i,k,t})$$

### Model specifications

We estimated model parameters using Markov Chain Monte Carlo (MCMC) methods. For each model we used uninformative priors and ran two chains for 30,000 iterations, discarding the first 20,000 runs as burn-in and thinning by 2. We ran all models using package “R2jags” in Program R (3.3.1) [37]. We assessed convergence by visually inspecting model trace plots and confirming that values for the potential scale reduction factor were <1.1 for all model parameters [38]. We identified a covariate effect as important if the 95% credible interval (CRI) for the posterior mean of the parameter coefficient did not overlap zero [34]. We also generated estimates for two derived parameters: the estimated number of occupied sites each year and the annual occupancy-based population growth rate *λ* [34].

## Results

**Fig 2:**
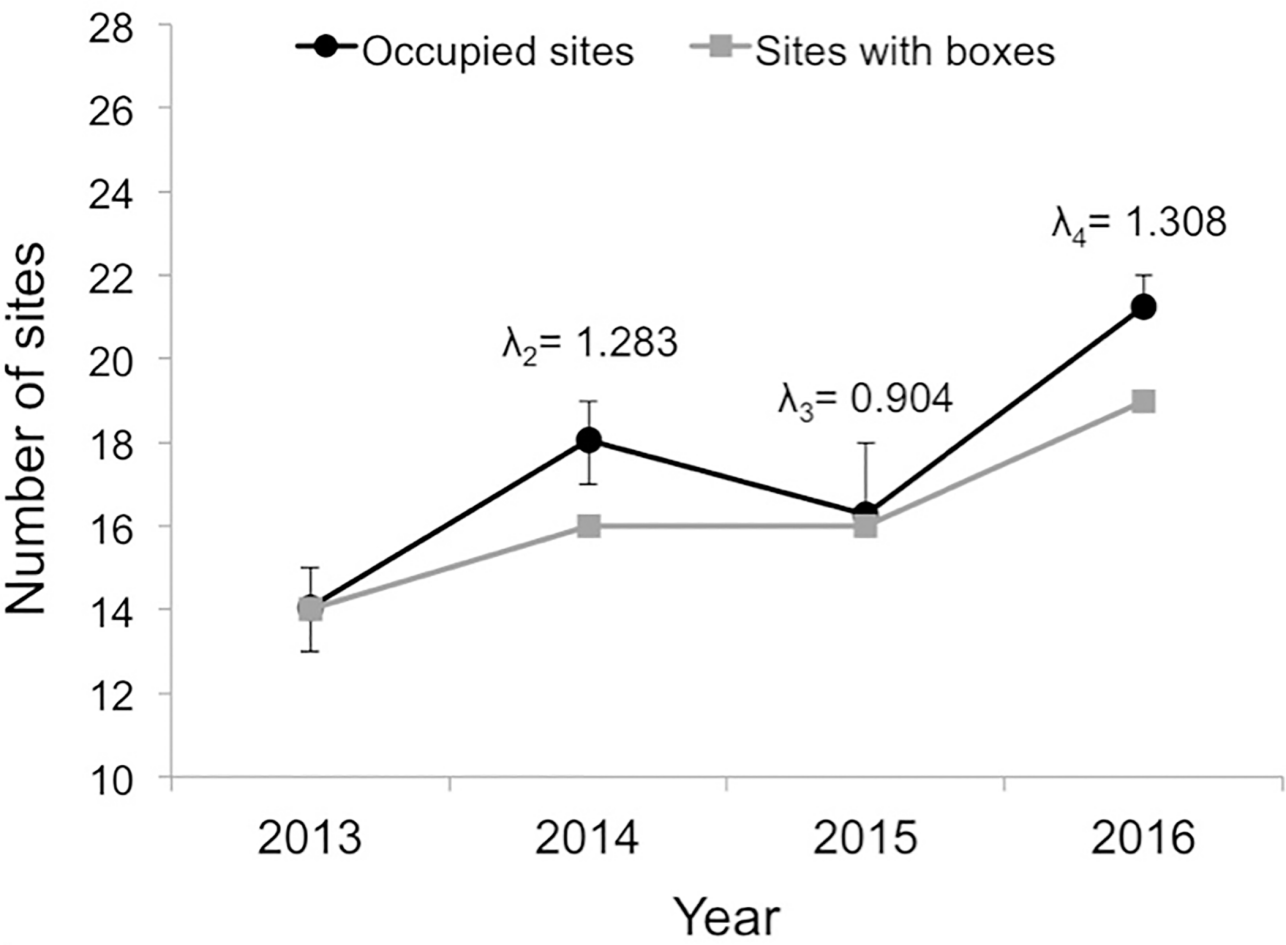
Number of sites with nest boxes and mean estimates of number of sites occupied by kestrels between 2013–2016. Error bars indicate 95% credible intervals. Occupancy-based population growth rates λ_2–4_ refer to changes since previous year.

Over the 4 years of this study, we detected kestrels at 22 of the 28 survey sites and during 133 out of 1176 total surveys. We detected kestrels at sites along all 4 transects; however, we observed kestrels along the western transect in 2016 only, after we had installed nest boxes during the fall of 2015 (Fig 1). The number of sites occupied by kestrels increased between 2013 and 2016, as the number of boxes we installed increased, with positive occupancy-based population growth occurring between 2013 and 2014, as well as between 2015 and 2016 (Fig 2).

**Fig 3:**
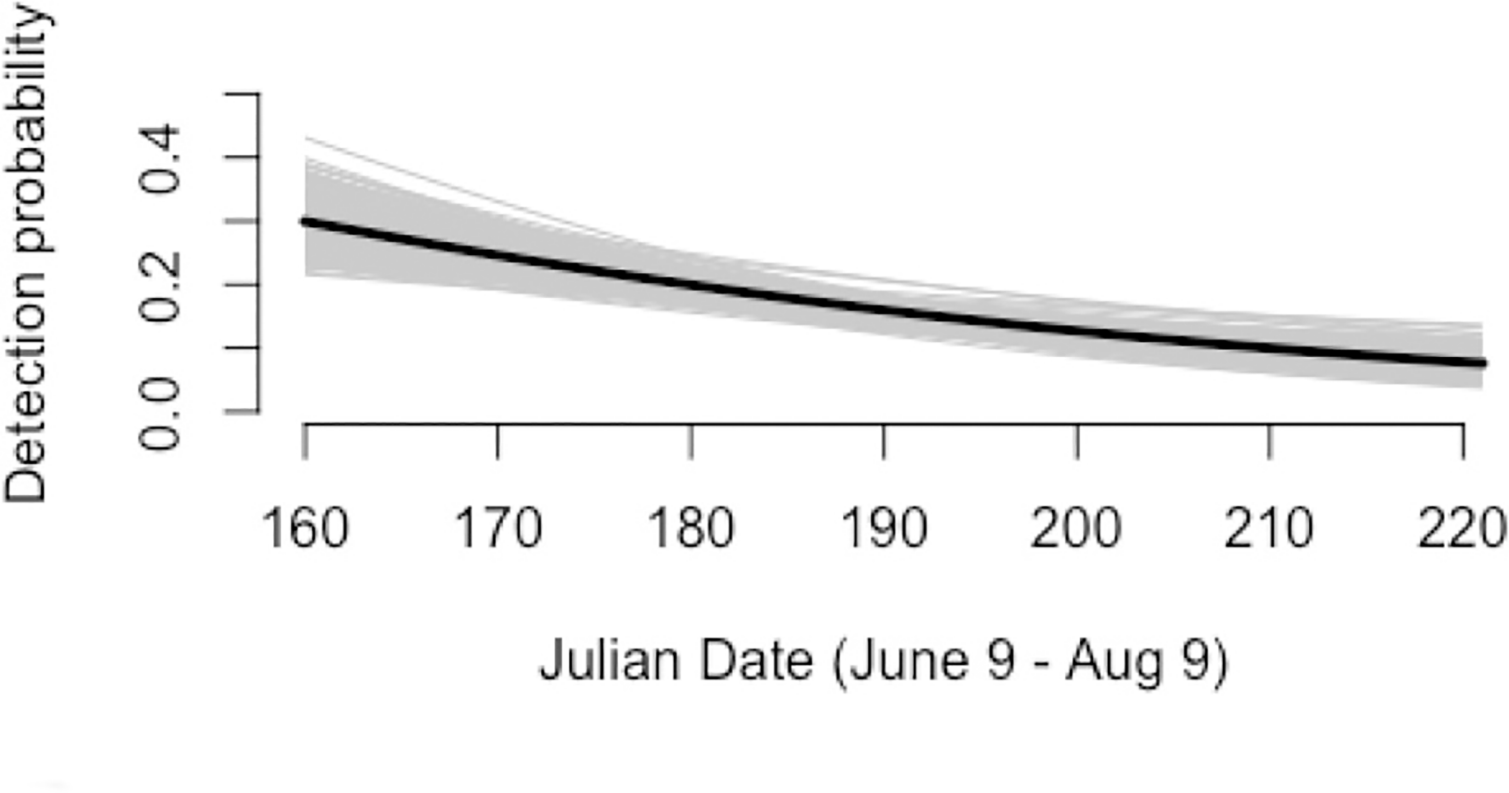
Predictions of the relationship between Julian date and kestrel detection probability *p*. Black line shows posterior mean, and gray lines show the relationship based on a random posterior sample of size 200 to visualize estimation uncertainty [34].

The covariate coefficient for *site box* in all ecological process models had 95% CRIs that did not overlap zero, indicating that nest boxes were an important predictor of first-year site occupancy, site colonization, and site persistence (Table 1). Furthermore, *neighbor box* had an important effect on site colonization: sites were more likely to become occupied if an adjacent site had nest boxes. The random effect of *transect* did not appear to have an important effect. Only *date* had an important effect on detection probability: kestrel detectability decreased between June and August (Fig 3).

**Table 1:** Multi-season occupancy modeling results for kestrel presence.

				95% CRI	
Parameter		Mean	SD	2.5%	97.5%
*ψ* (first year occupancy)	α0 (intercept)	2.16	3.63	-4.94	8.69
	**α1 (*site box*)**	**7.88**	**1.67**	**3.88**	**9.93**
	α2 (*neighbor box*)	1.04	2.73	-4.17	6.53
*γ* (colonization)	α3 (intercept)	2.15	3.61	-4.96	8.49
	**α4 (*site box*)**	**5.45**	**2.68**	**0.85**	**9.78**
	**α5 (*neighbor box*)**	**5.25**	**2.63**	**0.92**	**9.73**
*φ* (persistence)	α6 (intercept)	3.74	3.20	-2.30	9.50
	**α7 (*site box*)**	**7.62**	**1.88**	**3.09**	**9.91**
	α8 (*neighbor box*)	0.22	3.38	-6.96	6.82
*p* (detection)	β0 (intercept)	-1.64	0.11	-1.87	-1.43
	**β1 (*date*)**	**-0.43**	**0.10**	**-0.64**	**-0.23**
	β2 (*time*)	-0.09	0.098	-0.28	0.052
	β3 (*survey*)	-0.138	0.097	-0.33	0.10
	β4 (*nest distance*)	0.036	0.097	-0.15	0.23
	*transect*				
	Western	-0.066	1.37	-3.15	2.79
	Eastern	-0.66	1.38	-4.33	1.22
	Northern	0.87	1.93	-1.02	5.54
	Southern	1.41	2.4	-2.76	5.82

Posterior summaries for intercepts, covariate coefficients, and random effect coefficients from the multi-season models for kestrel first-year site occupancy, site probability, site colonization, site persistence, and detection probabilities. Important covariate effects are indicated in bold (95% CRI does not overlap zero) [34].

## Discussion

As predicted, kestrel presence was more likely at sites with nest boxes; furthermore, kestrels were more likely to colonize and persist at sites with nest boxes. Adding orchard nest boxes to additional sites in 2013 and 2015 resulted in an overall increase in kestrel presence between 2013 and 2016. Most notably, the western transect had no sites with nest boxes and no observed or model-estimated kestrel presence during the 2013–2015 seasons; however, we observed kestrels in 2016 following the installation of boxes. Combined with the high rates of orchard nest box use in this region [30], these results indicate that the local kestrel population is indeed limited by nest site availability. Previous research has shown that habitat quality is the primary driver of occupancy dynamics [39]. Our results support this finding and indicate that improving habitat quality at the territory level can increase the occupancy and persistence of local populations by increasing the likelihood of colonization of potential territories and decreasing the extinction risk for those territories.

Additionally, as predicted, sites neighboring those with boxes were more likely to become colonized, probably due to home ranges overlapping the two sites or a conspecific attraction effect causing kestrels to settle at sites neighboring occupied sites. As a result, the number of occupied sites in 2014 and 2016 exceeded the number of sites with boxes (Fig 2). However, the lack of an important effect of *neighbor box* on site persistence suggests that boxes in neighboring sites do not have as strong of an influence on kestrel site occupancy as boxes in the site itself. One explanation for the lack of an important effect of *neighbor box* on site persistence is that the sizes and shapes of kestrel home ranges likely vary each year. Thus, a site without boxes that is colonized by kestrels using a new nest box in the neighboring site may not remain occupied the following year if the kestrel pair using the box has a smaller or differently-shaped home range that does not include the neighboring site. Given the strong relationship between the presence of nest boxes at a site and kestrel site occupancy, installing additional boxes at the sites that still lack them would be necessary to potentially ensure kestrel presence at all sites, regardless of kestrel pair home range sizes. Future work is therefore needed to determine the saturation point for increasing kestrel presence using nest boxes in this region.

Julian date had a negative effect on kestrel detectability; kestrel detections decreased between June and August. Previous research [40] has shown a decrease in Eurasian Eagle-Owl (*Bubo bubo*) detections over the breeding season for unsuccessful breeders only, which the authors attributed to a decrease in conspicuous territorial behaviors after reproductive failure. Because kestrels using the new nest boxes during this study showed a 91% success rate, the decrease in detections observed during this study is more likely due to another source of behavioral change rather than reproductive failure. An alternative explanation is that kestrels are less conspicuous later in the breeding season due to lower hunting activity, as we predicted. Prey consumption requirements for breeding kestrels increase when the eggs hatch because the adults must provision their young [41], and while prey requirements for the family remain high after fledging, the adults cease provisioning the young within 3 weeks after fledging [42]. Thus, adult kestrel hunting activity is likely highest during the brood rearing period (June–July in our study) and may decrease during the postfledging period (July—August). The decrease in detections during the postfledging period may have also resulted from adults dispersing along with fledglings during the postfledging period prior to migration [32]. Without information on adult movements during the postfledging period, we cannot conclude whether our assumption of population closure was violated.

None of the remaining covariates had important effects on kestrel detectability. The lack of an effect of *time* suggests that kestrels are equally conspicuous in the morning and afternoon. This conclusion is supported by previous observations that kestrels hunt throughout the day without apparent peaks in activity [41]. The lack of an effect of *survey* suggests that observer presence during the initial survey did not affect kestrel detectability during the return survey, thus any potential temporal dependence between surveys is not the result of a behavioral response to the observer. Finally, *site distance* did not have an important effect, which indicates that detections were not biased towards kestrel activity at boxes with active nesting attempts within 250 m of the transect. This conclusion is further supported by the fact that we made no kestrel sightings at a nest box. The results from the detectability model indicate that kestrel researchers have flexibility in the timing of their surveys during the day and the placement of survey sites with regard to nest boxes.

Although kestrels are conspicuous open-habitat raptors, the occupancy model estimated our detection probabilities as <0.4 (Fig 3). These detection probabilities are lower than those reported from occupancy models for Peregrine Falcons [43], California Spotted Owls [44], and Cooper’s and Sharp-shinned Hawks [45]. The first explanation for the low detectability of kestrels during our study is that kestrels were not always within the 1.6 km × 500 m survey site areas and therefore were not available for detection during all surveys [36]. Another explanation is that we conducted passive surveys and did not lure kestrels using either prey [44] or broadcasts of conspecific calls [45].

## Study limitations

Our results support the conclusion that nest boxes determined the presence of kestrels at our study sites; however, our study design and modeling framework do no allow us to conclude that the breeding kestrel population has increased as a direct result of nest box installation. It is possible that the installation of nest boxes in the survey sites drew in breeding kestrels that had used other territories previously; the true nature and extent of nest site-limitation cannot be determined without also monitoring other sources of cavities throughout the region [24]. Nevertheless, our results provide strong evidence that the breeding kestrel population in this region is either limited by the number of nest sites available or prefers nest boxes for breeding.

Our survey design also included logistical constraints; we maximized the spatial and temporal replicates needed for occupancy modeling at the possible expense of introducing unresolved spatial and temporal dependence between sites and surveys. However, we included additional variables in our ecological and observation models to attempt to identify and explain possible biological sources for any dependence. We found that adjacent sites were dependent in that sites neighboring those with nest boxes were more likely to be colonized by kestrels than sites neighboring those without boxes. We also did not find an important difference in detections among initial and return surveys, which suggests that observer presence during the initial survey did not affect kestrel detectability during the return survey; however, the pairs of surveys could still be dependent in other ways. Nevertheless, we do not expect that temporal dependence significantly biased our detectability estimates because we observed all possible combinations of detection and nondetection for pairs of initial and return surveys (i.e. 00, 11, 01, and 10).

### Conclusions

Although our results do not directly correspond to an increase in the breeding kestrel population, our results indicate that kestrels in this fruit-growing region are more likely to choose breeding territories with nest boxes. Given the high reproductive success observed at nest boxes in this region [30], we therefore expect that orchard nest boxes can benefit the conservation of breeding kestrels in fruit-growing regions. Furthermore, increasing kestrel presence in and around orchards could enhance the ecosystem services provided by kestrels. Previous studies of raptors in agroecosystems have focused mainly on Barn Owls (*Tyto alba*) and their diets [46]; ongoing work therefore aims to examine the effects of kestrel presence and predation on prey abundances in orchards.

In addition, our territory-level results are highly relevant to the farmers and landowners on whose properties the boxes were installed, for we can conclude that installing a nest box in an orchard resulted in a high probability of kestrels occupying that orchard or the areas adjacent to it. Our results could encourage additional farmers to install and maintain nest boxes in fruit-growing regions where agricultural practices create open hunting habitat for kestrels that are therefore limited in these habitats primarily by nest site availability. Ongoing work aims to further inform box installation practices by exploring the relationship between the number of boxes installed at a site and kestrel presence in a fruit-growing region with lower nest box occupancy rates than observed in this region. Two major issues with nest box programs that can potentially be detrimental to kestrel populations are placement of boxes in low quality habitat and the installation of boxes that are not monitored or maintained [24]. Thus, installation of nest boxes in appropriate habitats and continued monitoring of these boxes following installation is important for effective nest box programs, and encouraging landowner engagement and investment in the boxes is important for the sustainability of nest box programs.

## Supporting information

S1 File Kestrel survey data. (TXT)

S2 File R and WinBUGS/JAGS code for occupancy model. (R)

## References

[1] Green RE, Cornell SJ, Scharlemann JPW, Balmford A. Farming and the fate of wild nature. Science 2005;307: 550–555. doi: 10.1126/science.1106049 15618485 10.1126/science.1106049

[2] Flynn DFB, Gogol-Prokurat M, Nogeire T, Molinari N, Richers BT, Lin BB, et al Loss of functional diversity under land use intensification across multiple taxa. Ecol Lett. 2009;12: 22–23. doi: 10.1111/j.1461-0248.2008.01255.x 19087109 10.1111/j.1461-0248.2008.01255.x

[3] Jiménez-Franco MV, Martínez JE, Calvo JF. Lifespan analyses of forest raptor nests: patterns of creation, persistence and reuse. PloS ONE. 2014;12: e0093628 10.1371/journal.pone.0093628 PMC3981714 24717935

[4] Newton I. The role of nest sites in limiting the numbers of hole-nesting birds: A review. Biol Conserv. 1994;70: 265–276.

[5] Heneberg P. Burrowing bird’s decline driven by EIA over-use. Resour Policy. 2013;38: 542–548.

[6] Gibbons P, Lindenmayer DB, Fischer J, Manning AD, Weinberg A, Seddon J, et al The future of scattered trees in agricultural landscapes. Conserv Biol. 2008;22: 1309–1319. doi: 10.1111/j.1523-1739.2008.00997.x 18680500 10.1111/j.1523-1739.2008.00997.x

[7] Wiebe KL. Nest sites as limiting resources for cavity-nesting birds in mature forest ecosystems: a review of the evidence. J Field Ornithol. 2011;82: 239–248.

[8] Robles H, Ciudad C, Matthysen E. Responses to experimental reduction and increase of cavities by a secondary cavity-nesting bird community in cavity-rich Pyrenean oak forests. For Ecol Manage. 2012;277: 46–53.

[9] Arlettaz R, Schaub M, Fournier J, Reichlin TS, Sierro A, Watson JEM, et al From publications to public actions: When conservation biologists bridge the gap between research and implementation. BioScience 2010;60: 835–842.

[10] Kiss O, Elek Z, Moskát C. High breeding performance of European Rollers *Coracias garrulus* in a heterogeneous farmland habitat of southern Hungary. Bird Study. 2014;61: 496–505.

[11] Paz A, Jareño D, Arroyo JL, Viñuela J, Arroyo B, Mougeot F, et al Avian predators as a biological control system of common vole (*Microtus arvalis*) populations in north-western Spain: Experimental set-up and preliminary results. Pest Manag Sci. 2013;69: 444–50. doi: 10.1002/ps.3289 22517676 10.1002/ps.3289

[12] Whelan CJ, Wenny DG, Marquis RJ. Ecosystem services provided by birds. Ann N Y Acad Sci. 2008;1134: 25–60. doi: 10.1196/annals.1439.003 18566089 10.1196/annals.1439.003

[13] Maas B, Karp DS, Bumrungsri S, Darras K, Gonthier D, Huang JC-C, et al Bird and bat predation services in tropical forests and agroforestry landscapes. Biol Rev. 2015;91: 1081–1101. doi: 10.1111/brv.12211 26202483 10.1111/brv.12211

[14] Jedlicka JA, Greenberg R, Letourneau DK. Avian conservation practices strengthen ecosystem services in California vineyards. PloS ONE. 2011;6: e27347 doi: 10.1371/journal.pone.0027347 22096555 10.1371/journal.pone.0027347 PMC3212556

[15] Mols CMM, Visser ME. Great Tits (*Parus major*) reduce caterpillar damage in commercial apple orchards. PloS ONE. 2007;2: e202 doi: 10.1371/journal.pone.0000202 17285148 10.1371/journal.pone.0000202 PMC1784073

[16] Kay BJ, Twigg LE, Korn TJ, Nicol HI. The use of artificial perches to increase predation on house mice (*Mus domesticus*) by raptors. Wildlife Res. 1994;21: 95–105.

[17] Kross SM, Tylianakis JM, Nelson XJ. Effects of introducing threatened falcons into vineyards on abundance of passeriformes and bird damage to grapes. Conserv Biol. 2012;26: 142–149. doi: 10.1111/j.1523-1739.2011.01756.x 22010952 10.1111/j.1523-1739.2011.01756.x

[18] Smallwood JA, Bird DM. American Kestrel (*Falco sparverius*) In: Poole A, Gill F, editors. The Birds of North America 602. Washington DC: American Ornithologists’ Union; 2002.

[19] Shave ME. Evaluating the conservation and agricultural applications of orchard nest boxes for a declining raptor. Ph.D. Thesis, Michigan State University. 2017. Available from: https://gradworks.umi.com/10/27/10275213.html.

[20] Farmer CJ, Smith JP. Migration monitoring indicates widespread declines of American Kestrels (*Falco sparverius*) in North America. J Raptor Res. 2009;43: 263–273.

[21] Smallwood JA, Causey MF, Mossop DH, Klucsarits JR, Robertson B, Robertson S, et al Why are American Kestrel (*Falco sparverius*) populations declining in North America? Evidence from nest box programs. J Raptor Res. 2009;43: 274–282.

[22] Sauer JR, Hines JE, Fallon J, Pardieck KL, Ziolkowski DJ Jr., Link WA. The North American Breeding Bird Survey, results and analysis 1966–2013. Version 01.30.2015. U.S.G.S. Patuxent Wildlife Research Center; 2014 Available from: http://www.mbr-pwrc.usgs.gov/bbs.

[23] J Rohrbaugh r. RW, Yahner RH. Effects of macrohabitat and microhabitat on nest-box use and nesting success of American Kestrels. Wilson Bull. 1997;109: 410–423.

[24] McClure CJW, Pauli BP, Heath JA. Simulations reveal the power and peril of artificial breeding sites for monitoring and managing animals. Ecol Appl. 2017;27: 1155–1166. doi: 10.1002/eap.1509 28117915 10.1002/eap.1509

[25] Smallwood JA, Collopy MW. Southeastern American Kestrels respond to an increase in the availability of nest cavities in north-central Florida. J Raptor Res. 2009;43: 291–300.

[26] Askham LR. Effect of artificial perches and nests in attracting raptors to orchards. In Davis LR, Marsh RE, editors. Proceedings of the 14th Vertebrate Pest Conference. Davis: University of California Davis Press; 1990. pp. 144–148.

[27] Brown JL, Collopy MW, Smallwood JA. Habitat fragmentation reduces occupancy of nest boxes by an open-country raptor. Bird Conserv Int. 2014;24: 364–378.

[28] USDA 2012 Census of Agriculture. County profile: Leelanau County, Michigan. United States Department of Agriculture National Agricultural Statistics Service; 2014 Available from: http://www.agcensus.usda.gov/Publications/2012/Online_Resources/County_Profiles/Michigan/cp26089.pdf.

[29] Brewer T, McPeek GA, Adams RJ Jr.. The atlas of breeding birds of Michigan East Lansing: Michigan State University Press; 1991.

[30] Shave ME, Lindell CA. American Kestrels occupying nest boxes in Michigan cherry orchards show high reproductive rates and tolerance of monitoring. J Raptor Res. 2017;51: 50–60.

[31] Smallwood JA, Winkler P, Fowles GI, Craddock MA. American Kestrel breeding habitat: The importance of patch size. J Raptor Res. 2009;43: 308–314.

[32] Olea PP. Postfledging dispersal in the endangered Lesser Kestrel Falco naumanni. Bird Study. 2001;48:110–115.

[33] Saracco JF, Siegel RB, Wilkerson RL. Occupancy modeling of Black-backed Woodpeckers on burned Sierra Nevada forests. Ecosphere. 2011;2:art31.

[34] Kéry M, Schaub M. Bayesian Population Analysis Using WinBUGS. New York: Elsevier; 2012.

[35] Palmer RS. Handbook of North American birds. Volume 5 New Haven: Yale University Press; 1988.

[36] Schmidt JH, McIntyre CL, MacCluskie MC. Accounting for incomplete detection: what are we estimating and how might it affect long-term passerine monitoring programs? Biological Conservation. 2013;160:130–139.

[37] Su Y-S and Yajima M. R2jags: Using R to Run 'JAGS'. R package version 0.5–7. 2015; Available from: https://CRAN.R-project.org/package=R2jags.

[38] Gelman A, Carlin JB, Stern HS, Rubin DB. Bayesian Data Analysis, Second Edition Boca Raton: Chapman & Hall; 2003.

[39] Robles H, Ciudad C. Influence of habitat quality, population size, patch size, and connectivity on patch-occupancy dynamics of the middle spotted woodpecker. Conserv Biol; 26:284–293. doi: 10.1111/j.1523-1739.2011.01816.x 22268847 10.1111/j.1523-1739.2011.01816.x

[40] León-Ortega M, Jiménez-Franco MV, Martínez JE, Calvo JF. Factors influencing territorial occupancy and reproductive success in a Eurasian Eagle-owl (*Bubo bubo*) population. PloS ONE. 2017;12: e0175597 doi: 10.1371/journal.pone.0175597 28399175 10.1371/journal.pone.0175597 PMC5388503

[41] Balgooyen TG. Behavior and ecology of the American Kestrel (*Falco sparverius*) in the Sierra Nevada of California. University of California Publications in Zoology 1976;103: 1–83

[42] Varland DE, Klaas TM. Development of foraging behavior in the American Kestrel. J Raptor Res. 1991;25: 9–17.

[43] Bruggeman JE, Swem T, Andersen DE, Kennedy PL, Nigro D. Multi-season occupancy models identify biotic and abiotic factors influencing a recovering Arctic Peregrine Falcon (*Falco peregrinus tundrius*) population. Ibis. 2015;158: 61–74.

[44] MacKenzie DI, Nichols JD, Seamans ME, Guitierrez RJ. Modeling species occurrence dynamics with multiple states and imperfect detection. Ecology. 2009;90: 823–835. 19341151 10.1890/08-0141.1

[45] Carlson JE, Piirto DD, Keane JJ, Gill SJ. Estimating site occupancy and detection probabilities for Cooper’s and Sharp-shinned Hawks in the southern Sierra Nevada. J Raptor Res. 49: 450–457.

[46] Labuschagne L, Swanepoel LH, Taylor PJ, Belmain SR, Keith M. Are avian predators effective biological control agents for rodent pest management in agricultural systems? Biol Control. 2016;101: 94–102.

